# Supplementary material for: Identification and expression analysis of S-alk(en)yl-L-cysteine sulfoxide lyase isoform genes and determination of allicin contents in Allium species
Source: PLoS One. 2020 Feb 24;15(2):e0228747. doi: 10.1371/journal.pone.0228747 (PMC7039512; doi:10.1371/journal.pone.0228747)
Supplement: S1 Data — (ZIP) [file pone.0228747.s001.zip › Certification letter United Kingdom.pdf]

EDITORIAL CERTIFICATE LETTER

This document is to certify that the manuscript listed below was edited for proper English language, grammar, punctuation, spelling, and overall style by one of the highly qualified subject-expert native English speaking editors at **NativeEnglishEdit.com**

The substantive content of the article mentioned below remains the full responsibility of the author/authors:

TITLE OF ARTICLE:
IDENTIFICATION AND EXPRESSION ANALYSIS OF S-ALK(EN)YL-L-CYSTEINE SULFOXIDE LYASE ISOFORM GENES AND DETERMINATION OF ALLICIN CONTENTS IN ALLIUM SPECIES

AUTHOR(S):
VAHID SAYADI, GHASEM KARIMZADEH, SAJAD RASHIDI MONFARED, MOHAMMAD REZA NAGHAVI

REFER CODE:
EE-2019-33338407-SAYADI-3689 TECH

Native English Edit
www.NativeEnglishEdit.com

Documents receiving this certification should be English-ready for publication; however, the author has the ability to accept or reject our suggestions and changes.
This certificate may be verified at:
www.NativeEnglishEdit.com
London
East End Road 27, N 3 3QT
United Kingdom
