# Supplementary material for: Identification and expression analysis of S-alk(en)yl-L-cysteine sulfoxide lyase isoform genes and determination of allicin contents in Allium species
Source: PLoS One. 2020 Feb 24;15(2):e0228747. doi: 10.1371/journal.pone.0228747 (PMC7039512; doi:10.1371/journal.pone.0228747)
Supplement: S1 Data — (ZIP) [file pone.0228747.s001.zip › EE-2019-33338407-Sayadi-3689 TECH - Edited.docx]

# Identification and expression analysis of *S-alk(en)yl-L-cysteine sulfoxide lyase* isoform genes and determination of allicin contents in *Allium* species

Vahid Sayadi^1^, Ghasem Karimzadeh^1*^, Sajad Rashidi Monfared^2^, & Mohammad Reza Naghavi^3^

Department of Plant Genetics and Breeding, Faculty of Agriculture, Tarbiat Modares University, Tehran, Iran

2 Department of Agricultural Biotechnology, Faculty of Agriculture, Tarbiat Modares University, Tehran, Iran

3 Agronomy and Plant Breeding Department, Agricultural College, University of Tehran, Karaj, Iran

^*^ Corresponding author

E-mail: karimzadeh_g@modares.ac.ir

# Abstract

Alliinase is the key enzyme in allicin biosynthesis pathway. In the current study, the identification and sequencing of alliinase genes along with determination of allicin contents were reported for *Allium* species with a novel report for Iranian endemic species, with the presence of different isoforms in the *Allium* being discovered for the first time. In bulbs tissue, the highest allicin concentration was in *Allium sativum*, *A.* *umbilicatum*, and *A. fistolosum* (1.185%, 0.367%, and 0.34%, respectively), followed by *A. spititatum* (0.072%), *A. lenkoranicum* (0.055%), *A. atroviolaseum* (0.36%), *A. rubellum* (0.041%), and *A. stamineum* (0.007%). The highest allicin content in the leaves and roots were in *A. sativum* (0.13%), and *A. stamineum* (0.195%), respectively. The ORFs length ranged from 1416 in *A. sativum* (*iso-alliinase2*; *ISA2*) to 1523 bp in *A. sativum* (*alliinase*); the identity with *A. sativum* (*alliinase*) varies from 95% to 68% for *A. ampeloprasum,* and *A. sativum* (*iso‑alliinase1*, *ISA1*) respectively. These data suggested that both *ISA1* and *ISA2* had a high expression in the roots and bulbs compared to *A. sativum* as the control in all species. Note that ISA1 and ISA2 were not expressed in the leaves. The results showed that isoforms expression patterns among different tissues in *Allium* species were variable. The presence of various isoforms is a possible explanation for the difference between the species in terms of obtained results, especially the amount of allicin.

**Keyword:** *Allium* species; *Alliinase* isoforms*, Iso-alliinase*, *Alliinase* sequencing, Allicin.

# Introduction

*Allium* L. is one of the largest genera in the family of the Amaryllidaceae, encompassing over 900 species [1,2]. The main center of its diversity is central Asia, including the territory of Iran and the Mediterranean, while the second distribution center for garlic and many *Allium* is western North America [3–6]. *Allium* species are primarily found in temperate, semi-arid, and arid regions of the northern hemisphere [7]. The results of recent classifications propose 15 subgenera and 56 sections for *Allium* [5], from which more than 30 species, including several endemics, grow in Iran [8]. The flavor of freshly chopped *Allium* species, such as garlic, is due to the alliin lyase enzyme activity (also known as alliinase or S-alk(en)yl-L-cysteine sulfoxide lyase). The major organosulfur components in *Allium* species are alliin and isoalliin [9]. In an intact cell, alliin exists in the cytoplasm and lies there while being physically separated from alliinase which is stored in the vacuoles [10,11]. In garlic, alliinase was first described by Stoll and Seebeck in 1947 [12]. Alliin is a natural substrate of alliinase (EC 4.4.1.4). The enzyme hydrolyzes it to pyruvate, ammonia, and allicin [13]. Allicin is unstable and it quickly decomposes into other compounds, such as diallyl disulfide [10,12,14]. It is an organosulfur compound which plays some roles in the prevention and treatment of diseases [15]. It has also a variety of antimicrobial activities and medical roles, such as reducing cholesterol, triglyceride, lowering blood pressure, stimulating the immune system, plus antifungal, anticancer, antioxidant, and anti-inflammatory effects [16–18]. On the other hand, alliinase is the key enzyme in allicin biosynthesis pathway. Alliinase is an important enzyme which is a part of the plant defense against pathogenic microbes and herbivores.. It is a protein that exists in all tissues of garlic [19]. The domestic *Allium* (such as onion and, garlic, chives, and leek) contains high concentrations of organic sulfur compounds especially in their bulbs and leaves. In the parenchymatous bundle sheaths of garlic, alliinase comprises up to 10–12% of the soluble clove protein material. Alliinase characterization has been studied from some *Allium*s, such as *A. sativum, A. cepa*, *A. tuberosum,* and *A. ascalonicum* [20–24]. Less work has been devoted to the phylogenetic analysis and the protection of wild *Allium* species as garlic relatives [25–27]. It has been reported that some *Allium* extracts contain allicin, with many studies reporting that the amounts of allicin in *Allium* extracts vary in different regions [10–13,28]. In the current report, alliinase genes have been identified and sequenced, and the allicin contents have been determined for *Allium* species with a novel report for Iranian endemic species. Finally, the presence of different isoforms in the *Allium* has been discovered for the first time.

# Results and Discussion

# Identification and characterization of alliinase isoforms

A fragment of approximately 1500 bp of the alliinase gene was successfully amplified with the new primers, using cDNA as template. The sequences of fragments were deposited in the NCBI GenBank Database (Table 1; Fig 1).

**Table 1- Information of the identified alliinase gene from the different species**

| Species | | Accession number | ORF length  (bp) | G+C content  (%) | Identity^a^ (%) |
| --- | --- | --- | --- | --- | --- |
| *A. sativum* | *Alliinase* | FJ786257 | 1523 | 43.3 | 100 |
|  | *ISA1* | MH492261 | 1494 | 41.4 | 68 |
|  | *ISA2* | MH492262 | 1416 | 39.3 | 69 |
| *A. fistolosum* | | MF972076 | 1443 | 42.7 | 91 |
| *A. umbilicatum* | | MH021987 | 1440 | 42.7 | 92 |
| *A. lenkoranikum* | | MH021989 | 1440 | 42.5 | 92 |
| *A. rubellum* | | MH021988 | 1440 | 43.5 | 91 |
| *A. ampeloprasum* | | MG742366 | 1449 | 43.1 | 95 |
| *A. ascalonicum* | | MG742367 | 1440 | 41.2 | 91 |
| *A. chinensis* | | MG742368 | 1440 | 41.4 | 91 |
| *A. macrostemon* | | MG742369 | 1428 | 42.2 | 70 |
| *A. tuberosum* | | MG742370 | 1434 | 41.1 | 85 |

^a^ with *A. sativum* (*alliinase*) as a control

**Fig 1. Positions of nested-PCR primers, and the expected lengths of PCR products, containing *Alliinase* gene. Arrows and lines represent primers and PCR products of each primer pairs, respectively.**

In this study, alliinase gene was scrutinized and two *iso-alliinase* genes (*ISA1* and *ISA2*) were investigated. The ORF length of gene sequences ranged from 1416 in *A. sativum* (*ISA2*) to 1523 bp in *A. sativum* (*Alliinase*). The G+C content of the analyzed alliinase genes ranged from 39.3% (in *A. sativum* - *ISA2*) to 43.5% (in *A. rubellum*). The nucleotide sequences from all *Allium* species were aligned and compared to alliinase amino acid sequences of *A. sativum* (control). Identity with *A. sativum* (*Alliinase*) was 95% for *A. ampeloprasum*, 92% for *A. umbilicatum* and *A. lenkoranikum*, 91% for *A. ascalonicum*, *A. chinensis*, *A. fistolosum* and *A. rubellum,* 85% for *A. tuberosum,* 70% for *A. macrostemon,* 69% and 68% for ISA2 and ISA1 (in *A. sativum*) respectively (Table 1). There is an EGF-like domain in the N-terminal part of the alliinase structure; these domains are small disulfide-rich structures in a conserved form (a polypeptide with ~ 50 amino-acid residues long) [29–31]. Frequently, EGF-like domain constitutes modules for binding to other proteins. They are often unusual in plant proteins found in the secreted proteins [30–32]. Among plant enzymes, alliinase is an example of a catalytic domain fused to an EGF-like domain. Note that the sequence alignment of complete alliinase sequences from different species shows a strictly conserved pattern (C–x18–19–C–x–C–x2–C–x5–C–x6–C, Fig 2).

**Fig 2. Sequence alignment of the N-terminal segments of *iso-alliinases* (ISA) genes. Black letters on gray background, residues of conserved cysteine residues in EGF-like domain.**

This pattern has been known in different species and is reported by some researchers in *Allium* species [30–34]. The functional role of EGF-like domain in alliinases is unclear. Its possible role is associated with the vacuolar localization of alliinase, where one may speculate that it may act as a binding site for other proteins or a hypothetical alliinase receptor. A phylogenetic tree of alliinase was constructed from different plants using MEGA7.0 based on CLUSTALW2 alignments. The results revealed that alliinase from *A. tuberosum, A. chinensis, A. fistolosum, A. ascalonicum, A. cepa, A. umbellicatum, A. umpeloperasum, A. sativum (alliinase), A. rubellum, A. lenkoranicum* was grouped into one cluster, while *A. macrostemon*, *A. sativum (ISA1)*, and *A. sativum (ISA2)* were classified into another cluster (Fig 3).

**Fig 3. Phylogenetic analysis of identified *Allium*. *iso-alliinase* (*ISA*) and other alliinase proteins from various species, using Mega7.InK software, through the maximum likelihood method. Protein accession numbers are as: *A. cepa*: AAA32639.1 and *A. sativum* (alliinase): ACN78838.1. Numbers on the branches represented bootstrap support for 1000 replicates.**

## Allicin contents

The allicin concentration of eight *Allium* species was analyzed using HPLC; the results for each tissue (bulbs, leaves, and roots) are shown in fig 4.

**Fig 4. Allicin content and relative expression of *iso-alliinase* gene (*ISA*) in eight *Allium* species, including *A. sativum, A. stipitatum, A. atroviolaceum, A. fistolosum, A. umbellicatum, A. lenkoranicum, A. stamineum*, and *A. rubellum*. Error bars represent SE (n = 3).**

All activities were done quickly as allicin is extremely unstable and instantly decomposed into organosulfur compounds such as diallyl sulfide (DAS), diallyl disulfide (DADS), diallyl trisulfide (DAT), dithiins, and ajoene [35]. In bulb tissue, the highest allicin concentration was in *Allium sativum* (control), *A.* *umbilicatum*, and *A. fistolosum* (1.185%, 0.367%, and 0.34%, respectively), followed by *A. spititatum* (0.072%), *A. lenkoranicum* (0.055%), *A. atroviolaseum* (0.36%), *A. rubellum* (0.041%), and *A. stamineum* (0.007%; Fig 4). Further, allicin content in the leaves and roots for *A. sativum, A. stipitatum, A. atroviolaceum, A. fistolosum, A. umbellicatum, A. lenkoranicum, A. stamineum*, and *A. rubellum* was 0.13, 0.075, 0.053, 0.035, 0.047, 0.088, 0.057%, and 0 for leaves, and 0.052, 0.148, 0.037, 0.028, 0.076, 0.077, 0.195, and 0.001 for roots, respectively (Fig 4). For the first time, we report that the extracts from the whole Iranian endemic *Allium* contain allicin. Large variations in allicin contents were found across different parts (Fig 4).

The presence of the allicin precursor and its derivative products in green garlic extracts has also been reported [28,36–39]. In a study, in the quantification of the total thiosulfinate of different *Allium* spp. by HPLC analysis, *A. sativum* showed higher amounts of total thiosulfinate compared to the other species [40]. Allicin contents in the dry weight of garlic ranging from 2 to 4 mg g^–1^ have been reported by many researchers [39,41]. Wang *et al.* (2014) reported that the amounts of allicin ranged from 0.81 to 3.01% [42]. According to British Pharmacopoeia, the minimum allicin content, in order to ensure pharmaceutical and economic viability of garlic powder, is 4.5 mg g^-1^ [28,43]. It has been shown that the allicin content in *Allium* extracts varies considerably across different regions [28,44,45]. It is well known that the allicin content is reasonably variable, and based on the amount of allicin determined for *Allium* in this study (Fig 4), it may provide pharmacological effects in some *Allium* species.

**Relative expression analysis of alliinase genes**

The qPCR technique was applied to determine the relationship between the allicin content and the gene expression pattern of alliinase isoforms influencing the allicin content. The expression of alliinase genes from bulbs, leaves, and roots in eight species was also examined: *Allium lenkoranicum, A. atroviolaceum, A. fistolosum, A. stipitatum, A. sativum* (control)*, A. rubellum, A. stamineum*, and *A. umbellicatum*. The maximum levels of gene expressions of the *alliinase* gene in bulbs were detected in *A. umbilicatum* and *A. fistolosum* (~ 1.6 and 1.5-fold, respectively). However, *A. sativum* with a high content of allicin (1.185%) had a low gene expression level compared to *A. umbilicatum* and *A. fistolosum*. Furthermore, this condition occurred in the leaves and roots, where *A. umbilicatum*, *A. fistolosum*, and *A. lenkoranicum* with a low allicin content (0.047, 0.035, and 0.088% for leaves; 0.076, 0.028, and 0.077% for roots, respectively) had a higher *Alliinase* gene expression (~ 6.7, 7, and 4.6 fold for leaves; 11.3, 5.2, and 2.3 fold for roots, respectively) compared to *A. sativum -alliinase-* (Fig 4). The relative expression of alliinase gene in the leaf of *A. rubellum* was low (0.15-fold), while no allicin content was detected. Note that the alliinase expression varies among the bulbs, leaves, and roots of garlic. It has also been suggested that garlic root tissue expresses a distinct alliinase isozyme with very low homology to the bulb enzyme [21].

In the current study, the presence of *iso-alliinase* genes was identified. Accordingly, three isoforms of the enzymes were identified as *Alliinase*, ISA1, and ISA2. Primary data analysis for roots indicated that the gene expression level did not match and it was not consistent with allicin amounts across all species. Other findings revealed that the previously designed primers for *Alliinase* gene were not suitable and amplified both ISA1 and ISA2. To solve this failure, the design of primers was carried out according to the differences, specifically for each gene (Table 2).

**Table 2- Information and sequences of used primers in real time PCR and *alliinase* identification**

| Use | Gene | Primer | Sequence (5' → 3') |
| --- | --- | --- | --- |
| Nested – PCR | *Alliinase*  (Outside of ORF) | Forward1 | GGGAGATTATAAGGAAGTGGAAC |
|  |  | Reverse1 | TTAAATGAATGGACGGCGG |
|  | *Alliinase*  (Inside of ORF) | Forward. ORF | GTGACATGGAGTTTGAAGGC |
|  |  | Reverse. ORF | CGCTTCAACCATATCCTTCAAATAG |
|  | *Alliinase*  (Out /In.) | Forward1 | GGGAGATTATAAGGAAGTGGAAC |
|  |  | Reverse ORF | CGCTTCAACCATATCCTTCAAATAG |
|  | *Alliinase*  (In. / Out) | Forward ORF | GTGACATGGAGTTTGAAGGC |
|  |  | Reverse1 | TTAAATGAATGGACGGCGG |
|  | *Iso-Alliinase 1*  (Outside of ORF) | Forward1 | CAACAACACTACAAACGCAC |
|  |  | Reverse1 | CGACATATTCTTCACTCATCCC |
|  | *Iso-Alliinase 1*  (Inside of ORF) | Forward. ORF | TCCCTTCCTCAGACTATCC |
|  |  | Reverse. ORF | ATGAAAGGACGCTCGATTG |
|  | *Iso-Alliinase 2*  (Outside of ORF) | Forward1 | GACTAAACAAAGCGAAATAGAGG |
|  |  | Reverse1 | GTAGAGAACACACAGGTACAA |
|  | *Iso-Alliinase 2*  (Inside of ORF) | Forward. ORF | TAATCAGAGGTGGGGAAACT |
|  |  | Reverse. ORF | TAATAAGGCTCAACCGTGC |
| qRT-PCR | *β-Actin* | Forward | TTGCTGGTGATGATGCTCCAAG |
|  |  | Reverse | CCATGTCATCCCAGTTGCTCAC |
|  | *Alliinase*  (Inside of ORF) | Forward | ATGGGTGAAGTGTGAATGGGA |
|  |  | Reverse | AGTCTTCCTCTTCGCTTCAACC |
|  | *Iso-Alliinase 1* | Forward | CAATCGAGCGTCCTTTCATC |
|  |  | Reverse | CGACATATTCTTCACTCATCCC |
|  | *Iso-Alliinase 2* | Forward | TGAAGAGGTACTTAGAAATGGAC |
|  |  | Reverse | GTAGAGAACACACAGGTACAA |

Expression studies using qPCR indicated that the highest level of *ISA1* gene in bulbs was detected in *A. lenkoranicum* and *A. fistolosum* (~1229.9 and 1040.9 fold, respectively), followed by *A. rubellum* (271.79 fold), *A. umbilicatum* (55.7 fold), *A. atroviolaceum* (11.14 fold), *A*. *stipitatum* (3.08 fold), and *A. stamineum* (1.73 fold). These amounts for ISA2 were 4.37, 3.81, 12.35, 5.15, 8.85, 7.4, and 8.19 folds respectively (Fig 4). In the roots for ISA1 and ISA2 in *A. umbilicatum*, *A. fistolosum, A*. *stipitatum, A. lenkoranicum, A. stamineum, A. atroviolaceum*, and *A. rubellum*, the values were 1271.42 and 1405.45; 60.05 and 17.38; 101.84 and 21.06; 6.32 and 2.85; 4.7, and 3.03; 2.67 and 3.38; 1.01, and 6.17 fold, respectively (Fig 4). These data suggested that both *ISA1* and *ISA2* had a high expression in the roots and bulbs compared to *A. sativum* as the control in all species except for *A. rubellum*, such that , the expression amounts were lower relatively in bulb compared to the roots in all species. Note that ISA1 and ISA2 were not expressed in the leaves.

Rabinkov *et al*. (1994) described that alliinase cloned in *A. sativum* tissues was expressed in the bulbs and leaves with a high alliinase activity, but not in the roots [21]. This demonstrated the presence of a nonhomologous alliinase gene in *A. sativum* roots. The proteins of alliinase isoform from *A. sativum* (*ISA1* and *ISA2*) were coded by a cDNA with low sequence identity to other *Allium* alliinases. Regarding the roots of *A. sativum,* Rabinkov *et al*. (1994) reported a protein with low sequence homology to *A. sativum* alliinase cDNA, but with an alliinase activity [21]. Alliinase in *A. cepa* is active as a trimer and tetramer [45–47]. Van Damme *et al.* (1997) reported that in some *Allium* spp., alliinase has been shown to aggregate with low molecular-mass lectins into stable active complexes [48], while Lancaster *et al*. (2000) found that multimeric forms did not aggregate in the onion root [47].

In some *Allium*s, it has been reported that the root alliinase has a wider C-S lyase activity. Expression studies using RNA and northern analysis showed that *A. cepa* root alliinase cDNA was expressed to a far greater extent in roots than in leaves and bulbs confirming that the alliinase cDNA of leaves was not expressed in roots [47]. Note that the expression is inconsistent at different growth stages of plant. Low alliinase activity in the seeds of *A. cepa* (cv. Rijnsburger) was reported by Freeman (1975) which was less than 2% of that in bulbs, and increased rapidly during the seedling development to reach a stable maximum, 15 to 20 days post-germination [48].

## Conclusion

This study aimed to investigate the *Allium* species, especially some Iranian endemic *Allium* species in terms of allicin contents, and to identify as well as analyze the expression of *alliinase* isoform genes. The results showed that allicin contents and *alliinase gene* expression levels in the *Alliums* were highly variable (Fig 4). The production of secondary metabolites varied among the species of a genus as well as its ecotypes, due to the ecological conditions and the gene expression levels [28,49–51]. To eliminate the environmental effects, the plants were propagated under the same conditions. Eliminating environmental effects via plant propagation under the same conditions have been noted by others studies [42,52,53]. Some researchers have announced that genetic factors are more likely to be influential than geographical origins in the allicin production potential [28]. Meanwhile, it was shown that allicin content in *Allium* extracts is variable across various regions [28,44,54]. An aim was to investigate the potential of various species in terms of gene expression and allicin contents. Further, differences between the species and the possible reasons were studied. In the current study, the presence of three different isoforms in the *Allium* was discovered for the first time. These differences in our findings can be presumably attributed to the presence of the isoforms. Regarding the novelty, there has been an emphasis on Iranian species; nevertheless, there has been no report on the presence of three isoforms across different tissues (bulb, leaf, and root) of the *Allium* species. The data of the identified *Alliinase*, *ISA1* and *ISA2* genes with their accession numbers are presented in Table 1. Numerous factors affect the production of secondary metabolites in plants such as genotype, plant genetic, plant physiology, environmental and ecological conditions [49,51,52,55,56]. Note that in comparison with simple pathways, the complex metabolic pathways were affected by more regulatory elements . In other words, fewer variable factors such as lower number of genes affect the end product in a simple network. The complexity of the pathway affects the metabolite production rate. In this regard, alliinase hydrolyzes alliin to allicin [13]. The biosynthetic pathway to alliin is still not clear [57]. Alliinase is the key enzyme in allicin biosynthesis pathway. In addition to the complexity of the biosynthetic route, genes expression levels and enzyme activities also affect the allicin contents. Most studies have shown a close relationship between metabolome and genes expression levels [51,52,56–60]. The results showed that the expression patterns of isoforms were variable among different tissues in *Allium* species (Fig 4). The presence of various isoforms is a possible explanation for the difference between the species in terms of obtained results, especially the amount of allicin.

It has been suggested that two different alliinase isoforms are present in *A. sativum*, one of which is specific for 1-PECSO (Trans-(+)-S-(1-propenyl)-L-cysteine sulphoxide) and alliin, while the other is specific for MCSO ((+)-S-Methyl-L-cysteine sulphoxide) [41]. It has been reported that the enzyme isoform was inactive in some *Allium* species during in situ test for alliinase activity in the IEF gel [47]. Lancaster *et al.* (2000) reported that two isoforms of alliinase existed in the onion root, where isoform 1 had pI = 9.3, while isoform 2 had pI = 7.6, 7.9, 8.1, and 8.3 [47]. They stated that both alliinase isoforms (I and II) showed similar enzymatic activity across the range of substrates. In contrast, only C-S lyases with Cys sulfoxide lyase activity have been reported for *Alliums* [61]. Lancaster *et al.* (2000) declared that the isoform I could not be sequenced. On the other hand, the identity between Alliinase, ISA1 and ISA2 with the isoform II of onion root alliinase was 54.30, 81.1, and 64.85 respectively. In the sequence from other *Allium* alliinases, *A.* *cepa* root alliinase has wider C-S lyase activity. *A. cepa* root alliinase may have a function in sulfur assimilation and remobilization in roots [47].

In the current study, the identification and sequencing of alliinase genes along with determination of allicin contents were reported for *Allium* species with a novel report for Iranian endemic species, with the presence of different isoforms in the *Allium* being discovered for the first time. Further studies such as enzyme assay for measuring the enzymatic activity across the different species, studying x-ray crystallography and the molecular dynamics of proteins can also be beneficial to obtain more details. Indeed, although this study intended to enhance our knowledge about the *Allium* species, further studies are still required to clarify the details. More studies contribute to a better understanding of the gene expression pattern, e.g. examining different growth stages (i.e., initial stage, vegetative growth, reproductive phase and, maturation stage) or under growth conditions (i.e., changes in temperature, soil composition and light). According to our research, the amino acid sequences of alliinases described here displayed a substantial similarity with other known alliinases. These findings are in accordance with other results from different papers suggesting that the alliinase gene displays a high variability among different species. Thus, it cannot be used as a phylogenetic marker; however, it can easily discriminate between closely related species [62].

# Materials and Methods

*Allium lenkoranicum, A. atroviolaceum, A. fistolosum, A. stipitatum, A. sativum, A. rubellum, A. stamineum*, and *A. umbellicatum* were collected from the natural habitats across various geographic locations of Iran (Table 3).

**Table 3- Local information of studied *Allium* species**

| Species | IBRC ^a^ No | Local collection sites | Latitude (N) Longitude (E) | Altitude (m) |
| --- | --- | --- | --- | --- |
| *A. sativum* | - | Bahar, Hamadan, Iran | 34° 55′ 48° 26′ | 1722 |
| *A. fistolosum* | - | Ghalea Now-e Ghar, Tehran. Iran | 35° 50′ 51° 50′ | 1189 |
| *A. stipitatum* | P1010429 | Mian Mil, Kermanshah, Iran | 34° 36′  47° 46′ | 2526 |
| *A. umbilicatum* | P1009439 | Kangelu, Alborz, Iran | 35° 50′ 51° 03′ | 1340 |
| *A. lenkoranikum* | P1008766 | Chashm, Semnan, Iran | 35° 57′ 53° 08′ | 1570 |
| *A. rubellum* | P1009972 | Qutor, Western Azerbaijan, Iran | 38° 36′ 44° 40′ | 1524 |
| *A. ampeloprasum* | P1009877 | Payam, East Azerbaijan, Iran | 38° 19′ 45° 47′ | 2220 |
| *A. ascalonicum* | - | Changsha, Hunan, China | 28° 13′ 112° 56′ | 48 |
| *A. chinensis* | - | Changsha, Hunan, China | 28° 13′ 112° 56′ | 48 |
| *A. macrostemon* | - | Changsha, Hunan, China | 28° 13′ 112° 56′ | 48 |
| *A. tuberosum* | - | Changsha, Hunan, China | 28° 13′ 112° 56′ | 48 |

^a^ Iranian biological resource center

To eliminate the environmental effects, the collected bulbs were maintained and propagated under the same conditions as in Iranian Biological Resource Center (IBRC), Tehran, Iran. The sampled plants at the immature stage were frozen in liquid nitrogen and then stored at -80 °C until further analysis.

## Determining the amount of allicin

### HPLC system and chromatographic conditions

The HPLC system consisted of Agilent 1100 HPLC Series system (Agilent, Santa Clara, CA, USA). Its analyses were performed on a C18 column (250 mm × 4.6 mm) where the allicin was detected at 254 nm wavelength by a UV–visible detector. Methanol and water (50:50%, v/v) were used as mobile phase with a flow rate of 0.7 ml min^-1^ at ambient temperature. The final injection volume was 20 μl. The allicin content was evaluated according to the method described in British pharmacopoeia with some modifications [28,43].

**Preparation of the *Allium* extracts and internal standard**

Butylparahydroxybenzoate was used as the internal standard (IS) for the quantification of allicin which was prepared in the mobile phase (20 mg in 100 ml). Half of the materials collected from five plants, were directly frozen in liquid nitrogen and powdered, using mortar and pestles. Further, 0.8 g powder was homogenized with 20 ml distilled water and sonicated for 5 min continuously at 100% amplitude, using an ultrasonicator (Elmasonic s30, Germany) in an ice container, and then incubated for 30 min at 25 °C. The obtained mash was poured through a five-layer cheesecloth and allow to drain, then transferred into 50 ml falcon tube. The extracts and cell debris were separated by centrifugation (6000 g) for 20 min at 4 °C. The supernatant was transferred into a new sterile 50 ml falcon tube. Also, 10 ml supernatant was diluted to 25 ml by adding mixture A (solution of anhydrous formic acid (1%; v/v): methanol (HPLC grade), 40:60), and centrifuged at 6000 g for 5 min at 4 °C. Further, 0.5 ml of IS was diluted to 10 ml with a supernatant of the second centrifugation in a volumetric flask. Note that allicin is unstable at high temperatures and the assay must be carried out as quickly as possible. Thus, the sample solutions were stored at -70 °C, before injection [14].

### Determination of allicin

The following equation was used to calculate the amount of allicin in the samples [28,43].

Allicin (%) = S_1_m_2_ × 22.75 S_2_m_1_ where;

m_1_ = mass of the garlic powder (0.8 g),

m_2_ = mass of butyl parahydroxybenzoate (0.02 g) in IS solution,

S_1_ = area of the peak corresponding to allicin,

S_2_ = area of the peak corresponding to internal standard.

## Assembly of reads and identification of alliinase gene

The plants were separated into bulbs, leaves, and roots. Half of each tissue (bulb, leaf, and root) was cut and mixed for RNA extraction and expression analyses, while the other half was considered for allicin measurement. Alliinase gene for *A. ascalonicum, A. chinensis, A. macrostemon*, and *A. tuberosum* was predicted through the NCBI Sequence Read Archive (SRA, http://www.ncbi.nlm.nih.gov/sra). SRA accession numbers were SRX1560692, SRX1560673, SRX1560658, and SRX1560563, respectively. Read sequences for desired *Allium* were downloaded from several RNAseq projects (http://www.ncbi.nlm.nih.gov/sra/). Then, more than 400 individual reads for each *Allium* with substantially similar reads were selected, using the Offline BLAST Software v.2.7.0 [63]. After assembling the read sequences, using Codon Code Aligner v. 5.0.1. Program, the consensus sequences for assembled reads were created. Next, an ORF was found for each chosen consensus sequence via ORF finder (http://www.ncbi.nlm.nih.gov/gorf/gorf.html). Finally, gene-specific PCR primer pairs were designed for PCR-amplification of alliinase genes, based on the complete ORF cDNA sequences (Table 2). The primers were confirmed by the Oligo Analyzer v.3.1 (eu.idtdna.com/calc/analyzer) and NCBI/Primer-blast (www.ncbi.nlm.nih.gov/tools/primer-blast/index.cgi?LINK_LOC=BlastHome). For predicting the alliinase isoforms, different contigs resulting from assembling *Allium sativum* reads sequences (SRA accession number: SRX3055368) were used for further analysis after which the primers were designed based on the obtained desired contigs (Table 2).

## RNA extraction and cDNA Synthesis

Total RNA was extracted using Ribospin^TM^ Plant kit (GeneAll Biotechnology Co., Ltd., Songpa-gu, South Korea) according to the manufacturer’s instructions. The RNA samples were treated with Qiagen RNase-Free DNase (Qiagen, 79254, Qiagen Inc., Midland, ON, Canada) for 30 min at 37 °C to remove the genomic DNA. Following the manufacturer’s protocol, 1 µg of total RNA was used to make cDNA at a volume of 20 µl, using Thermo Scientific Revert-Aid™ First-Strand cDNA Synthesis Kit (Fermentas, K1622, Thermo Fisher Scientific, Hudson, NH, USA).

## Nested PCR detection and phylogenetic construction

The nested primers for the amplification of alliinase genes were designed based on different positions on consensus sequences to amplify the untranslated region (UTR) and coding region of alliinase gene fragments overlapping with each other to confirm the characterized ORFs (Table 2 and Fig 1). The expected length of alliinase was amplified by RT-PCR from first-strand cDNA from leaves, using Hyperscript™ RT PCR master mix (GeneAll Biotechonolgy Co. Ltd, Fig 1). PCR was carried out with newly specific primers for *alliinase* gene in three repeats. The PCR program was as follows: 95°C for 3 min, followed by 34 cycles at 95 °C for 5 min, 55 °C for 30 S, 72°C for 1.30 min, with the final elongation of 10 min at 72 °C. PCR products were separated in 1% (w/v) agarose TAE gel. The amplified PCR products of long fragments were cleanup by gel recover kit (Top Gel Recovery Kit, TOPAZ GENE RESEARCH., Cat. No.: TGK1006, Iran), and subjected to direct sequencing by an automatic sequence and dye-termination sequencing system (Macrogen Co., Seoul, South Korea). The sequences were edited and assembled by employing SeqMan (DNAstar) [64]. Also, the identification of open reading frames (ORFs) and conserved domains, as well as translated protein sequences were done using the BLASTN, BLASTP, ORF finder, available at http://www.ncbi.nlm.nih.gov/, and Pfam, available at http://pfam.xfam.org/. After a BLASTP search on the NCBI database, alliinase protein sequences were selected from different species with more than 50% identity with the coding region of the consensus sequences. To determine the relationship between the identified alliinase and the protein downloaded from the BLASTP search, multiple alignments were run using web-based Clustal Omega program (https://www.ebi.ac.uk/Tools/msa/clustalo/). The maximum likelihood method in MEGA7 was done for phylogenetic tree construction and 1000 iterations were applied for calculating the bootstrap value [65].

## Real-time PCR assay

The designed primer pairs were specific for the amplification of β-actin used as reference genes (Table 2). The qPCR was performed using specific primers (Table 2) on a BioRad MiniOpticon real-time PCR detection system (Applied Biosystems, Foster City, CA, USA) with the fluorescent dye SYBR®Green Master Mix 2X (Ampliqon, A323402, Denmark) in accordance with the manufacturer’s instructions. Specifically, 1 µl of the first strand cDNA was used as a template in 20 μl reactions, including 10 μl SYBR®Green PCR Master Mix and 0.3 pmol of each primer. The qRT-PCR was run at 95 °C (15 min), 35 cycles at 95 °C (20 s), 59 °C (30 s), and 72 °C (30 s), followed by gradient: 60-95 °C (5 s). The dissociation stage was accomplished to determine the PCR product size and to detect possible primer dimers. Triplets of all samples were run, and the negative control of the Master Mix in addition to primers was performed in all qPCR runs. The relative expression levels were calculated using the 2^−ΔΔCT^ method [66,67].

# Acknowledgements

Authors gratefully acknowledge the support provided for this survey by the Tarbiat Modares University, Tehran, Iran.

# References

1. Kusterer J, Fritsch RM, Keusgen M. *Allium* species from central and southwest Asia are rich sources of marasmin. J. Agric. Food Chem*.* 2011; 59: 8289-8297.doi:10.1021/jf201052u

2. Herden T, Hanelt P, Friesen N. Phylogeny of *Allium* L. subgenus *Anguinum* (G. Don. ex W.D.J. Koch) N. Friesen (Amaryllidaceae). Mol. Phylogenet. Evol*.* 2016; 95: 79-93. doi:10.1016/j.ympev.2015.11.004

3. Etoh, T., Simon P. Diversity, fertility and seed production of garlic. In: Rabinowitch HD and Currah L, editor. *Allium* Crop Science: Recent Advances. CAB Intl., Wallingford, UK; 2002. pp. 101–117.

4. Garcia-Lampasona S, Martinez L, Burba JL. Genetic diversity among selected Argentinean garlic clones (*Allium sativum* L.) using AFLP (Amplified Fragment Length Polymorphism). Euphytica*.* 2003; 132, 115-119. doi: 10.1023/A:1024606004596

5. Friesen N, Fritsch RM, Blattner FR. Phylogeny and new intrageneric classification of *Allium* (Alliaceae) based on nuclear ribosomal DNA ITS sequences. Aliso. 2006; 22: 372-395. doi:10.5642/aliso.20062201.31

6. Jabbes N, Geoffriau E, Le Clerc V, Dridi B, Hannechi C. Inter simple sequence repeat fingerprints for assess genetic diversity of tunisian garlic populations. J Agric Sci. 2011; 3: 77-85. doi:10.5539/jas.v3n4p77

7. Fritsch, RM., Friesen N. Evolution, domestication and taxonomy. In: Rabinowitch HD and Currah L, editor. *Allium* Crop Science: Recent Advances. CABI Publ, Wallingford; 2002. pp. 5–30.

8. Wendelbo P. Alliaceae. In: Rechinger KH, editor. Flora Iranica No 76 Graz. 1971.

9. Benkeblia N, Lanzotti V. *Allium* Thiosulfinates : Chemistry , Biological Properties and their Potential Utilization in Food Preservation. Food j. 2007;2: 193–201. doi: 10.1021/jf60174a007

10. Lancaster JE, Collin HA. Presence of alliinase in isolated vacuoles and of alkyl cysteine sulphoxides in the cytoplasm of bulbs of onion (*Allium cepa*). Plant Sci Lett. 1981; 22: 169-176 . doi:10.1016/0304-4211(81)90139-5

11. Ellmore GS, Feldberg RS. Alliin lyase localization in bundle sheaths of the garlic clove (*Allium sativum*). Am. J. Bot*.* 1994; 81: 89-94. doi:10.2307/2445567

12. Stoll, A., Seebeck E. Alliin, the pure mother substance of garlic oil. Experientia. 1947; 3: 114–115.

13. Weiner L, Shin I, Shimon LJW, Miron T, Wilchek M, Mirelman D, *et al*. Thiol-disulfide organization in alliin lyase (alliinase) from garlic (*Allium sativum*). Protein Sci. 2009; 18: 196-205. doi:10.1002/pro.10

14. Mansor N, Herng HJ, Samsudin SJ, Sufian S, Uemura Y. Quantification and Characterization of Allicin in Garlic Extract. J Med Bioeng. 2016; 5: 24–27. doi:10.12720/jomb.5.1.24-27

15. Nwachukwu I, Slusarenko A, Gruhlke M. Sulfur and Sulfur Compounds in Plant Defence. Natural product communications. 2012; 7: 395-400 .

16. Isaacsohn JL, Moser M, Stein EA, Dudley K, Davey JA, Liskov E, *et al*. Garlic powder and plasma lipids and lipoproteins: a multicenter, randomized, placebo-controlled trial. Arch Intern Med. 1998; 158: 1189-1194. doi: 10.1001/archinte.158.11.1189

17. Su CC, Chen GW, Tan TW, Lin JG, Chung JG. Crude extract of garlic induced caspase-3 gene expression leading to apoptosis in human colon cancer cells. In Vivo (Brooklyn). 2006; 20: 85-90 .

18. Kim CH, Lee MA, Kim TW, Jang JY, Kim HJ. Anti-inflammatory Effect of *Allium* *hookeri* Root Methanol Extract in LPS-induced RAW264.7 Cells. 7 Cells. J Korean Soc Food Sci Nutr. 2012; 41: 1645-1648. doi:10.3746/jkfn.2012.41.11.1645

19. Wang J, Cao Y, Sun B, Wang C, Mo Y. Effect of ultrasound on the activity of alliinase from fresh garlic. Ultrason Sonochem. 2011; 18: 534-540. doi:10.1016/j.ultsonch.2010.09.008

20. Clark SA, Shaw ML, Every D, Lancaster JE. Physical characterization of alliinase, the flavor generating enzyme in onions. J Food Biochem. 1998; 22: 91-103 . doi:10.1111/j.1745-4514.1998.tb00233.x

21. Rabinkov A, Zhu XZ, Grafi G, Galili G, Mirelman D. Alliin lyase (Alliinase) from garlic (*Allium sativum*). Biochemical characterization and cDNA cloning. Appl Biochem Biotechnol. 1994; 48: 149-171. doi:10.1007/BF02788739

22. Manabe T, Hasumi A, Sugiyama M, Yamazaki M, Saito K. Alliinase [S-alk(en)yl-L-cysteine sulfoxide lyase] from *Allium* tuberosum (Chinese chive) purification, localization, cDNA cloning and heterologous functional expression. Eur J Biochem. 1998; 257: 21-30. doi:10.1046/j.1432-1327.1998.2570021.x

23. Van Damme E J M, Smeets K, Torrekens S, Leuven F, Van and Peumansi W J. Isolation and characterization of lectins and lectin-alliinase complexes from bulbs of garlic (*Allium sativum*) and ramsons (*Allium ursinum*). Glycoconj J. 1997; 14: 331-343. doi:10.1023/A:1018570628180

24. Schwimmer S. Characterization of S-propenyl-l-cysteine sulfoxide as the principal endogenous substrate of l-Cysteine sulfoxide lyase of onion. Arch Biochem Biophys. 1969; 130: 312-320 . doi:10.1016/0003-9861(69)90038-1

25. Rabinowitch, H. D. and OZ. Collection, preservation, characterization and evaluation of *Allium* species growing wild in Israel: Selected Examples. Wageningen, The Netherlands: Eucarpia, 3rd *Allium* Symposium; 1984. pp. 27-36.

26. Fritsch R. Taxonomy of the genus *Allium*: Contribution from IPK Gatersleben. Herbertia. 2001; 56: 19–50.

27. Keller ERJ, Senula A. Progress in structuring and maintaining the garlic (*Allium sativum*) diversity for the European genres project. Acta Horticulturae. 2001. 555: 189-193. doi:10.17660/ActaHortic.2001.555.28

28. Baghalian K, Ziai SA, Naghavi MR, Naghdi Badi H. Pre-planting evaluation of allicin content and botanical traits in Iranian garlic (*Allium sativum* L.) ecotypes. J Med Plants. 2005; 1: 50-59. doi:10.1016/j.scienta.2004.07.001

29. Campbell ID, Bork P. Epidermal growth factor-like modules. Curr Opin Struct Biol. 1993; 3: 385-392. doi:10.1016/S0959-440X(05)80111-3

30. Davis CG. The many faces of epidermal growth factor repeats. New Biol. 1990; 2: 410-419 .

31. Louie G V., Yang W, Bowman ME, Choe S. Crystal structure of the complex of diphtheria toxin with an extracellular fragment of its receptor. Mol Cell. 1997; 1: 67-78 . doi:10.1016/S1097-2765(00)80008-8

32. Otwinowski Z, Minor W. Processing of X-ray diffraction data collected in oscillation mode. Methods in Enzymology. 1997. 276: 307-326. doi:10.1016/S0076-6879(97)76066-X

33. Shimon LJW, Rabinkov A, Shin I, Miron T, Mirelman D, Wilchek M, *et al*. Two Structures of Alliinase from *Alliium sativum* L.: Apo Form and Ternary Complex with Aminoacrylate Reaction Intermediate Covalently Bound to the PLP Cofactor. J Mol Biol. 2007; 366: 611-625. doi:10.1016/j.jmb.2006.11.041

34. Bartholomeus Kuettner E, Hilgenfeld R, Weiss MS. The active principle of garlic at atomic resolution. J Biol Chem. 2002; 277: 46402-46407. doi:10.1074/jbc.M208669200

35. Brodnitz MH, Pascale J V., Van Derslice L. Flavor Components of Garlic Extract. J Agric Food Chem. 1971; 19: 273-275. doi:10.1021/jf60174a007

36. Kim MY, Chung SK. Analysis of nutritional and volatile flavor compounds of garlic shoot. Korean J Post-Harvest Sci Technol Agric Prod. 1997; 4: 61–68.

37. Edris AE, Fadel HM. Investigation of the volatile aroma components of garlic leaves essential oil. Possibility of utilization to enrich garlic bulb oil. Eur Food Res Technol. 2002; 214: 105-107. doi:10.1007/s00217-001-0429-2

38. Nasim SA, Dhir B, Samar F, Rashmi K, Mahmooduzzafar, Mujib A. Sulphur treatment alters the therapeutic potency of alliin obtained from garlic leaf extract. Food Chem Toxicol. 2009; 47: 888-892. doi:10.1016/j.fct.2009.01.024

39. Prati P, Henrique CM, Souza AS de, Silva VSN da, Pacheco MTB. Evaluation of allicin stability in processed garlic of different cultivars. Food Sci Technol. 2014; 3: 623-628. doi:10.1590/1678-457x.6397

40. Block E, Naganathan S, Putman D, Zhao SH. *Allium* chemistry: HPLC analysis of thiosulfinates from onion, garlic, wild garlic (ramsoms), leek, scallion, shallot, elephant (great-headed) garlic, chive, and Chinese chive. Uniquely high allyl to methyl ratios in some garlic samples. J Agric Food Chem. 1992; 40: 2418–2430. doi:10.1021/jf00024a017

41. Lawson LD, Hughes BG. Characterization of the formation of allicin and other thiosulfinates from garlic. Planta Med. 1992; 58: 345-350. doi:10.1055/s-2006-961482

42. Wang H, Li X, Shen D, Oiu Y, Song J. Diversity evaluation of morphological traits and allicin content in garlic (*Allium sativum* L.) from China. Euphytica. 2014; 198: 243-254. doi:10.1007/s10681-014-1097-1

43. Pharmacopoeia B. British pharmacopoeia commission. A-290. 2007; 4.

44. Arzanlou M, Bohlooli S. Introducing of green garlic plant as a new source of allicin. Food Chem. 2010; 120: 179-183. doi:10.1016/j.foodchem.2009.10.004

45. Schulz V. Garlic. In: Hansel, R., Tayler VE, editor. Rational Phytotherapy A Physicians’ Guide to Herbal Medicine. 3 rd. 1998. pp. 107–125.

46. Nock LP, Mazelis M. The C-S Lyases of Higher Plants : Direct Comparison of the Physical Properties of Homogeneous Alliin Lyase of Garlic (*Allium sativum*) and Onion (*Allium cepa*). Plant Physiol. 1987; 85: 1079-1083. doi:10.1104/pp.85.4.1079

47. Lancaster JE, Shaw ML, Joyce MD, McCallum J a, McManus MT. A novel alliinase from onion roots. Biochemical characterization and cDNA cloning. Plant Physiol. 2000; 122: 1269-1279. doi:10.1104/pp.122.4.1269

48. Freeman GG. Distribution of flavour components in onion (*Allium cepa* L.), leek (*Allium porrum*) and garlic (*Allium sativum*). J Sci Food Agric. 1975; 26: 471- 481. doi:10.1002/jsfa.2740260413

49. Darvishi Zeidabadi D, Jalali Javaran M, Dehghani H, Rashidi Monfared S, Baghizadeh A. An investigation of the HMGR gene and IPI gene expression in black caraway (*Bunium persicum*). 3 Biotech. 2018; 8: 405 . doi:10.1007/s13205-018-1404-y

50. Abedini D, Monfared SR, Abbasi A. The effects of promoter variations of the N-Methylcanadine 1-Hydroxylase (CYP82Y1) gene on the noscapine production in opium poppy. Sci Rep. 2018; 8: 4973 . doi:10.1038/s41598-018-23351-0

51. Salehi M, Karimzadeh G, Naghavi MR, Naghdi Badi H, Rashidi Monfared S. Expression of artemisinin biosynthesis and trichome formation genes in five *Artemisia* species. Ind Crops Prod. 2018; 112: 130-140 . doi:10.1016/j.indcrop.2017.11.002

52. Salehi M, Karim G, Naghavi MR, Badi HN. Expression of key genes affecting artemisinin content in five *Artemisia* species. 2018; 8: 12659. doi:10.1038/s41598-018-31079-0

53. Cortijo S, Aydin Z, Ahnert S, Locke JCW. Widespread inter-individual gene expression variability in *Arabidopsis thaliana*. Mol. Syst. Biol*.* 2019; 15: e8591. doi:10.15252/msb.20188591

54. Iberl B, Winkler G, Muller B, Knobloch K. Quantitative determination of allicin and alliin from garlic by HPLC. Planta Med. 1990; 56: 320-326. doi:10.1055/s-2006-960969

55. De Sousa Abreu R, Penalva LO, Marcotte EM, Vogel C. Global signatures of protein and mRNA expression levels. Molecular BioSystems. 2009; 5: 1512-1526. doi:10.1039/b908315d

56. Hamidi F, Karimzadeh G, Rashidi Monfared S, Salehi M. Assessment of Iranian endemic artemisia khorassanica: Karyological, genome size, and gene expressions involved in artemisinin production. Turkish J Biol. 2018; 42: 322-333. doi:10.3906/biy-1802-86

57. Borlinghaus J, Albrecht F, Gruhlke MCH, Nwachukwu ID, Slusarenko AJ. Allicin: Chemistry and biological properties. Molecules. 2014. doi:10.3390/molecules190812591

58. Rasouli O, Ahmadi N, Rashidi Monfared S, Sefidkon F. Physiological, phytochemicals and molecular analysis of color and scent of different landrace of Rosa damascena during flower development stages. Sci Hortic (Amsterdam). 2018; 231: 144-150. doi:10.1016/j.scienta.2017.12.010

59. Kakeshpour T, Monfared SR, Ebrahimi A, Beyraghdar Kashkooli A, Ebrahimie E. Expression analyses of salinity stress- associated ESTs in *Aeluropus littoralis*. Gene Expr Patterns. 2017; 25-26: 76-84. doi:10.1016/j.gep.2017.06.005

60. Mastakani FD, Pagheh G, Monfared SR, Shams-Bakhsh M. Identification and expression analysis of a microRNA cluster derived from pre-ribosomal RNA in Papaver somniferum L. and Papaver bracteatum L. PLoS One. 2018; 13. doi:10.1371/journal.pone.0199673

61. Ramírez EC, Whitaker JR. Cystine lyases in plants: A comprehensive review. Journal of Food Biochemistry. 1998; 22: 427-440. doi:10.1111/j.1745-4514.1998.tb00254.x

62. Drugǎ B, Şuteu D, Oana RC, Pârvu M, Dragoş N. Two novel alliin lyase (alliinase) genes from twisted-leaf garlic (*Allium obliquum*) and mountain garlic (*Allium* *senescens* ssp. montanum). Not Bot Horti Agrobot Cluj-Napoca. 2011; 39: 293-298. doi: 10.15835/nbha3926355

63. Altschul SF, Madden TL, Schäffer AA, Zhang J, Zhang Z, Miller W, *et al*. Gapped BLAST and PSI-BLAST: A new generation of protein database search programs. Nucleic Acids Research. 1997. 25: 3389-3402. doi:10.1093/nar/25.17.3389

64. Burland T. DNASTAR’s lasergene sequence analysis software. Methods Mol Biol. 2000;132: 71-91. doi: 10.1385/1-59259-192-2:71

65. Tamura K, Stecher G, Peterson D, Filipski A, Kumar S. MEGA6: Molecular Evolutionary Genetics Analysis Version 6.0. Mol Biol Evol. 2013; 30: 2725-2729. doi:10.1093/molbev/mst197

66. Livak KJ, Schmittgen TD. Analysis of relative gene expression data using real-time quantitative PCR and the 2^-ΔΔCT^ method. Methods. 2001; 25: 402-408. doi:10.1006/meth.2001.1262

67. Sehringer B, Zahradnik HP, Deppert WR, Simon M, Noethling C, Schaefer WR. Evaluation of different strategies for real-time RT-PCR expression analysis of corticotropin-releasing hormone and related proteins in human gestational tissues. Analytical and Bioanalytical Chemistry. 2005; 383: 768-775. doi:10.1007/s00216-005-0067-9
