# Supplementary figures and images for: Identification and expression analysis of S-alk(en)yl-L-cysteine sulfoxide lyase isoform genes and determination of allicin contents in Allium species
Source: PLoS One. 2020 Feb 24;15(2):e0228747. doi: 10.1371/journal.pone.0228747 (PMC7039512; doi:10.1371/journal.pone.0228747)

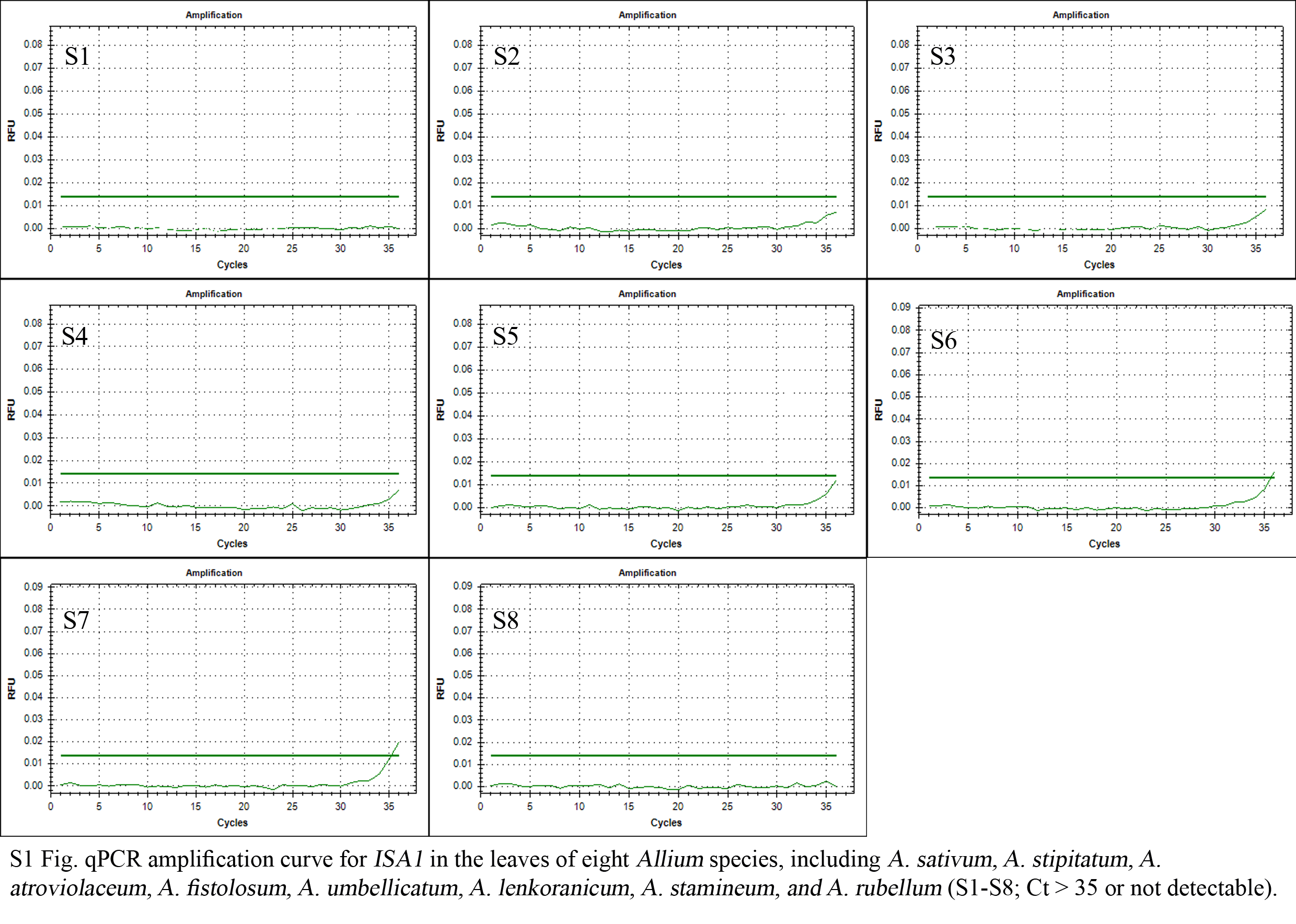

Supplement: S1 Fig — (TIF) [file pone.0228747.s002.tif]

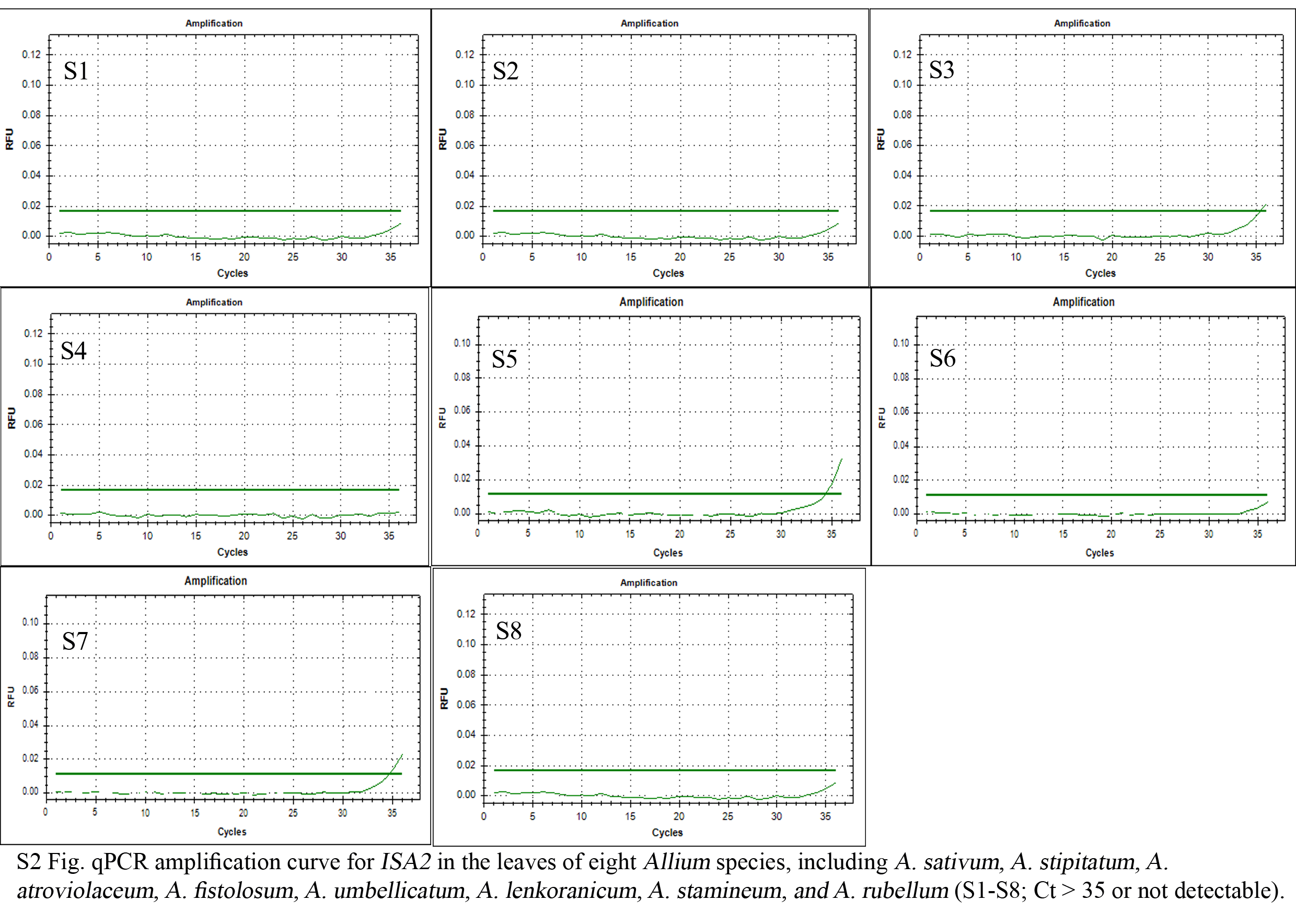

Supplement: S2 Fig — (TIF) [file pone.0228747.s003.tif]
